# Leveraging Academic-Medical Legal Partnerships to Advance Health Justice

**DOI:** 10.1017/jme.2024.5

**Published:** 2023

**Authors:** Vicki W. Girard, Yael Z. Cannon, Deborah F. Perry, Eileen S. Moore

**Affiliations:** 1: GEORGETOWN LAW CENTER, WASHINGTON, DC, USA; 2: GEORGETOWN UNIVERSITY, WASHINGTON, DC, USA; 3: GEORGETOWN UNIVERSITY SCHOOL OF MEDICINE, WASHINGTON, DC, USA

**Keywords:** Medical-Legal Partnership, Health Justice, Health Equity, Health Harming Legal Needs, Social Determinants Of Health, Interprofessional Education

## Abstract

Unmet legal needs contribute to housing, income, and food insecurity, along with other conditions that harm health and drive health inequity. Addressing health injustice requires new tools for the next generations of lawyers, doctors, and other healthcare professionals. An interprofessional group of co-authors argue that law and medical schools and other university partners should develop and cultivate Academic Medical-Legal Partnerships (A-MLPs), which are uniquely positioned to leverage service, education, and research resources, to advance health justice.

## Introduction

A patient with persistent and severe asthma attacks is struggling with a landlord who refuses to address the mold in her apartment that is exacerbating her asthma. As part of a medical-legal partnership, her doctor refers her to a lawyer, who provides the advocacy needed to enforce the landlord’s legal obligation to remediate the mold. Ultimately, with the support of her doctor and her lawyer, the landlord remediates the mold and the patient’s asthma improves.

Champions of health equity have long focused on the significant impact of negative social and political determinants of health on marginalized communities of color and vulnerable patient populations.^1^ When these drivers of health raise legal barriers, such as in the case of the asthma patient living with mold, they present as “health-harming legal needs” (i.e., unmet legal needs that negatively impact health and well-being).^2^ Areas where such needs commonly arise include housing, employment, food insecurity, education, insurance, public benefits, family stability, and immigration.

Medical-Legal Partnership (MLP) is a different approach to both healthcare and access to justice that brings providers and patients together with lawyers to address health-harming legal needs.^3^ By integrating legal care into healthcare, MLPs seek to help patients achieve optimal health and advance health justice more broadly.^4^ As a scholarly framework and a movement, health justice aims to leverage law and policy to dismantle systems of subordination that drive health inequities and build the power of individuals and communities to create and sustain conditions that support health and justice.^5^ Within that framework, MLPs are an important and innovative option for holistic problem-solving. By addressing the root causes of health disparities and other fundamental systemic inequities,^6^ MLPs advance health equity at the individual, systems, and population levels.^7^

Most commonly, these partnerships bring together legal services organizations with a hospital or other health system. Academic MLPs (A-MLPs), which have at least one university partner, have emerged as a special type of MLP. Often operating across and within various higher education sectors, A-MLPs share distinct service, education, and research features. How A-MLPs should leverage and maximize these features to advance health justice and participate in the next evolutionary stage of the MLP movement is of interest to the MLP field, and to the fields of health and law more broadly. Drawing on the experiences of the Georgetown University Health Justice Alliance (the HJA), other A-MLPs, current literature, and insights from the March 2023 conference, Academic Medical-Legal Partnerships: Equity, Evaluation, and Evolution (“A-MLP Conference”), this article is a first step in answering those questions.

Most commonly, these partnerships bring together legal services organizations with a hospital or other health system. Academic MLPs (A-MLPs), which have at least one university partner, have emerged as a special type of MLP.^8^ Often operating across and within various higher education sectors, A-MLPs share distinct service, education, and research features.^9^ How A-MLPs should leverage and maximize these features to advance health justice and participate in the next evolutionary stage of the MLP movement is of interest to the MLP field, and to the fields of health and law more broadly.^10^ Drawing on the experiences of the Georgetown University Health Justice Alliance (the HJA), other A-MLPs, current literature, and insights from the March 2023 conference, *Academic Medical-Legal Partnerships: Equity, Evaluation, and Evolution* (“A-MLP Conference”),^11^ this article is a first step in answering those questions.

Part I reports on the results of a national environmental scan^12^ conducted by the HJA and co-published with the National Center for Medical-Legal Partnership (National Center) defining A-MLPs and identifying their unique characteristics and contributions. Part II uses the scan, literature, and other resources to consider the academic community’s foundational rationales, objectives, and specific ideas for leveraging the unique features of A-MLPs. Part III builds on recent scholarship and momentum to examine how A-MLPs can contribute to the next evolutionary stage of MLPs and their efforts to advance health justice.

### I. The Academic-MLP

Across the United States, dozens of institutions of higher education house or are affiliated with an A-MLP. Many of these A-MLPs, consistent with the MLP movement generally, have grown organically, often at the impetus of dedicated faculty champions seeking to use the MLP model to achieve particular academic and health justice goals. Anecdotal evidence suggests, however, that without significant financial and other institutional support (including protected faculty time), it can be difficult for A-MLPs to reach their full academic, service, and research potential. Georgetown’s A-MLP champions knew that an ambitious cross-campus partnership would require a long-term commitment, financial and otherwise, from law and medical school leadership. In November 2016, a three-year memorandum of understanding was signed for funding, personnel, administrative, and other support to establish the HJA, with the Law Center also agreeing to fully fund the HJA Law Clinic, which launched in Fall 2017.

Without any formal definition or parameters for an MLP within an academic institution, articulating the benefits of MLP as an academic endeavor to university leaders, healthcare partners, faculty, alumni, development team members, and others was challenging. In response, the HJA decided to design and conduct the first national environmental scan of MLPs with evidence of engagement with a medical or law school to document their core elements, objectives, activities, and unique features.^13^ The intent was to define an “Academic MLP” and to lay the foundation for future A-MLP development and research. The HJA also aimed to acknowledge and promote others’ efforts to leverage their university settings to advance the broader MLP movement and health justice. The National Center supported the scan by sharing its prior MLP survey results and connections from the field.

The HJA supplemented the National Center’s baseline information with additional research of schools’ websites and other resources to identify 61 law school and 39 medical school participants. Outreach resulted in 39 law schools and 19 medical schools completing a web-based survey. In early 2020, semi-structured follow up interviews were conducted with 14 law schools and 5 medical schools, which were analyzed using MAXQDA and survey data with SPSS.

The scan results were framed around the National Center’s eight core MLP elements:^14^ a formal agreement, legal staffing, a defined population, a screening strategy, a lawyer in residence, healthcare team training by lawyers, information sharing, and a funding resource.^15^ The final report described how MLPs in higher education settings have adopted and adapted these eight elements and added three additional distinguishing features focused on education, learning environments, and research.^16^ Defining the “Academic MLP” as a distinct MLP type is intended, among other things, to build community and invite research and conversation to strengthen A-MLP efforts to advance health justice.

### II. Leveraging the Unique Features of A-MLPs to Advance Health Justice

Historically, MLPs have been born of necessity–emerging in response to specific health or law practice frustrations^17^ or to achieve particular patient or client focused goals.^18^ This genesis often forces health and legal professionals to work together in new ways and embrace new skills in the context of already busy practices, each with its own traditional and deeply rooted professional mores and routines.^19^ In contrast, A-MLPs more often start with an intent to engage specific learners early in their professional training and development, while their approaches to practice are still malleable.

The academic goals of A-MLPs significantly impact decisions about their structure, patient populations, legal services, and other programmatic elements, which are reflected in the national scan’s first two unique A-MLP features: (A) prioritizing pre-professional educational goals for learners; and (B) intentionally curating interprofessional learning environments for students to work across professions. The third feature, (C) a commitment to research and evaluation, stems from the special emphasis that universities place on such efforts and the resources they provide. The rationales, objectives, and ideas emerging from these three features provide a lens for analyzing of the current state of the A-MLP movement and can help guide its future directions and potential to advance health justice.

#### A. Prioritizing Pre-Professional Education

Consistent with their first feature, the prioritization of pre-professional education, A-MLPs center their work on students. These may include graduate and undergraduate students from law, medical, nursing, social work, public health, and other schools at different formational stages in their professional development.^20^ While a variety of other educational efforts and methods exist for teaching students about the social drivers of health, A-MLPs are unique in teaching learners how the law can improve patient and population health and giving them hands-on experience in identifying health-harming legal needs and addressing those needs through advocacy. MLPs have always had to make choices about legal service areas, patient populations, and partners in conjunction with geographic and other variables. Because, however, A-MLPs prioritize educating pre-professional learners, they must often be particularly strategic about how they deliver legal and health services to their communities while still meeting their pedagogical goals. A-MLPs are also increasingly teaching students how to use MLP to engage in structural law reform efforts^21^ and to work explicitly to advance racial justice.^22^

The value of the A-MLP for pre-professional education has been recognized at various law and medical schools, some of which have been engaged in MLP work for decades. Faculty and students at Georgia State, Brown, Yale, and Rutgers Universities, University of New Mexico, University of Michigan, Loyola University Chicago, and elsewhere have set examples for creatively using law and medical school platforms–sometimes in partnership and sometimes with different local health and legal providers–to engage pre-professional learners in MLP work.^23^ On the health side, one example of the benefits of reaching pre-professional learners is reflected in the common skill of taking a patient’s medical history. Medical students generally learn to ask about the basics of diseases, hospitalizations, medications, allergies, and a few standard lifestyle questions. They are also often taught about the negative impact that social determinants can have on health. Rarely, however, do they learn how to treat those determinants, especially where no tools are available to reduce their impact. Without training, students may naturally begin to categorize critical non-medical issues as falling into the domain of social work or patient navigation. When MLP learning is integrated into healthcare curricula, however, future health professionals learn to move beyond standard patient history questions and to delve more deeply into social determinants that may raise health-harming legal needs. By offering tangible solutions to issues like food and housing insecurity, A-MLPs empower future doctors and other health professionals to see themselves as more capable of addressing those barriers in ways that create more equitable opportunities for patients to achieve health and well-being.

Law school A-MLPs also train students to practice differently through approaches that have the potential to transform the legal profession, legal systems, and access to justice. Law schools are among the fastest growing A-MLP settings, and at least sixty-one currently evidence some level of MLP work (compared to 39 medical schools).^24^ In particular, A-MLPs are well-suited to clinical legal education, an experiential learning model with both practice and classroom components in which law students represent real clients under the close supervision of faculty.^25^ Many law school clinics provide free legal services to clients with low income as they seek to instill in law students the knowledge, skills, professionalism, values, and strategic expertise necessary to practice law effectively.^26^ Those with A-MLPs also teach students how the practice of law can facilitate access to justice that prioritizes prevention of legal and health crises and more holistic and interdisciplinary approaches to both justice and care.^27^

Across their different academic settings, A-MLPs equip future lawyers and health professionals to transform health and legal systems and to wield the law to pursue health justice. Students graduating with A-MLP experience may go on to transform their professional practices by helping them develop and prioritize holistic approaches to care that address the social and structural determinants of health driving racial health disparities in their communities. As learners build the knowledge, skills, behaviors, and values that will inform their careers, A-MLPs cultivate graduates who understand health-harming legal needs and are MLP practice-ready.^28^ Although not every graduate will join an MLP, exposing students to A-MLPs may generally foster an understanding of how law drives health disparities and a desire to champion health equity and justice throughout their careers and across different settings.^29^

#### B. Curating Interprofessional Learning Environments

Along with prioritizing pre-professional education for individual learners, A-MLPs rely on interprofessional education opportunities to deepen learning about how to work across disciplines to advance health justice.^30^ By engaging students during their academic careers, A-MLPs build the foundational expectation and expertise needed to take a holistic and collaborative approach to health and justice in the future. Some of that learning occurs naturally, by virtue of the interprofessional nature of all MLPs. A-MLPs however, specifically curate interprofessional environments (bringing together faculty, students, and professionals across disciplines) where students can both learn and teach, try new skills, and reflect on their efforts with appropriate oversight and feedback. Intentionally designing these places and spaces allows faculty to manage and enhance the impact of the interprofessional educational experience whether it happens in the classroom, through experiential programs or projects, or in clinical settings.

Among the most intensive interprofessional learning environments that A-MLPs offer students are real-world settings that engage them as impactful members of treatment teams providing wraparound services to patients.^31^ As aspiring lawyers and health professionals, these experiences allow students to grapple with a wide set of challenges facing marginalized and minoritized individuals and communities and to consider their responsibilities for addressing them.^32^ They may include opportunities to teach professionals of other disciplines, to help each other understand the power of the law to improve health and well-being for those who have experienced health inequities, and to develop advocacy strategies.^33^ One example of such a learning environment is the HJA Law Clinic, a key cornerstone of the HJA’s A-MLP. In the HJA Law Clinic, law students partner with fourth-year medical students in the School of Medicine’s Health Justice Scholars program, who spend 4-8 week formal rotations as full-time legal team members. Medical students apply their expertise in myriad ways, such as helping law students understand their clients’ diagnoses and collaborating with law students on client interviews to gain critical information, such as the health harms a client has experienced from substandard housing conditions. As law students collaborate with medical school faculty and students—as well as nursing, social work, and/or public health students and faculty—in seminar courses and on joint advocacy efforts, they all learn “to become effective practitioners who are sensitive to the connections between poverty, health, and law,” to recognize structural racism and other forms of discrimination as drivers of health equity, and to collaborate effectively across disciplines.^34^

Another HJA curated interprofessional opportunity engages learners in legislative advocacy. Over several months, teams of law and medical students prepare to meet with congressional representatives as part of an annual Capitol Hill Advocacy Day.^35^ As law students hone their legal advocacy skills, they learn how to engage and prepare healthcare professionals to participate in legislative and policy efforts. Similarly, as medical students share relevant healthcare knowledge and patient stories with A-MLP faculty and law students, they also learn to effectively use their expertise and voices to support advocacy aimed at advancing health justice. As with the HJA Law Clinic, this type of interprofessional learning illustrates how A-MLPs teach law students and their healthcare counterparts to identify and evaluate problems through a broader lens, looking at issues not as purely legal or medical, but untangling problems that often involve intertwined issues of health and injustice.^36^

Overall, A-MLPs can help enhance students’ problem-solving capacities as they process input from different disciplines and consider a range of tools that can advance holistic, client/patient-centered advocacy.^37^ As they come to recognize the connections between health inequities and injustice, the hope is that future lawyers will see their clients’ needs as more than legal, future healthcare professionals will see their patients’ needs as more than medical, and both will have developed the interprofessional collaboration skills to effectively address those needs.

#### C. Conducting Research and Evaluation

There is general consensus that the MLP model is effective, despite a limited number of rigorous empirical studies that systematically gather data over time on the impact of MLPs on a variety of outcomes.^38^ A-MLPs are uniquely positioned to leverage their university settings to forge interdisciplinary partnerships committed to the research and evaluation needed to examine and build the evidence base for the effectiveness of MLPs’ educational, service and policy work and determine the most effective approaches for health justice impacts. Although all MLPs engage in work that could contribute to the evidence base for the model, A-MLPs are more likely to have the research and publishing resources and incentive to pursue empirical research.

Designing research approaches to study the impacts of A-MLPs is a complex endeavor due to their multiple layers: patients/clients, learners, systems, and communities may all be affected by the partnerships. For A-MLPs, pre-professional learners are central to the narrative of how and to what extent MLPs are changing outcomes for the recipients of legal services. In MLP clinics, law students gain experience working with clients as part of an interprofessional team as they address the health-harming legal needs of specific patient populations under the supervision of law faculty. Working with their medical partners, the law students both learn and teach about the root causes of health inequities in the patients that become their clients. A-MLPs have the opportunity to measure law students’ changes in knowledge and publish this data as a part of their legal scholarship.^39^

More law schools than medical schools use the MLP model in clinical training. But medical education’s intense focus on assessing competencies at the undergraduate (i.e., the traditional 4-year medical school curriculum) and graduate (i.e., residency or fellowship) levels has resulted in more deliberate research focused on the impact of MLP on education and training around social determinants of health.^40^ Although the literature suggests a broad range of didactic and experiential efforts being made to teach medical learners at all levels about the social determinants of health, the impact of these efforts on actual healthcare practice or patient health remain unclear.^41^

The collaboration between fourth-year medical and law students in the HJA Law Clinic is an intensive interprofessional learning environment. It precipitated a longitudinal prospective cohort study and quasi-experimental design, the PIPEline Study,^42^ which refers to the idea that exposure to MLP as professional identities are forming might be preparing the next generation of health justice champions. At the onset, an articulated theory of change drove what outcomes were prioritized and the timing for measuring them. That theory posits that the interprofessional learning environments curated by the HJA will influence students’ knowledge and attitudes about people’s social needs and their beliefs about how law and policy can be used as a tool to address persistent barriers to health and well-being at the individual, systemic, and population levels. Student learning is also expected to reflect an appreciation for MLP as a collaborative healthcare delivery model, and their practical interprofessional experiential opportunities to increase their confidence and capacity to effectively contribute their legal advocacy expertise to the common endeavor of achieving health justice.

Designing research approaches to study the impacts of A-MLPs is a complex endeavor due to their multiple layers: patients/clients, learners, systems, and communities may all be affected by the partnerships.

In addition to measuring the impact of MLPs on student outcomes, A-MLPs are building the evidence base for the effectiveness of the model on patient/client, healthcare partner, and system-level outcomes.^43^ A recent scoping review intended to assess the peer-reviewed evidence for the effectiveness of MLPs reported 30 studies that met their inclusion criteria.^44^ The studies reported on a wide variety of outcomes, which provided support for the MLP model. Many of the studies focused on patient-level outcomes, with improvements in healthcare utilization, such as improved asthma and diabetes control and reduced hospitalizations being reported. Improvements in client housing conditions, access to utilities and other financial benefits were also cited. Other patient-level outcomes were reduced stress and anxiety, greater hope and empowerment, and higher levels of confidence and agency. About half of the studies noted changes in healthcare providers’ behaviors, such as increased screening for health-harming legal needs leading to referrals for legal services. One limitation of the scoping review was that many of the studies appeared to be purely descriptive in nature, failed to have a comparison group, and none appeared to be a randomized controlled trial.^45^ As the MLP field seeks to sustain their programs and to scale the approach to more settings, there is a need for A-MLPs to continue to build partnerships, especially with public health researchers and other researchers with expertise that may further scholarship in this area.

### III. Building on the A-MLP Momentum: Participating in the Next MLP Evolutionary Stage to Advance Health Justice

Health and legal systems in the United States have complicated histories that have caused, contributed, and continue to perpetuate many of the health, justice, and other inequities that harm minoritized and marginalized communities. A recurring theme at the A-MLP Conference and in recent scholarship is the responsibility that lawyers, physicians, nurses, public health professionals, social workers, and others engaged in MLP work have to help transform these systems, internally and structurally. Because A-MLPs operate at the intersections of interprofessional service, education, and research, they are well-poised to explore and assess how they can contribute to these objectives. Building on A-MLP scholarship and ideas from the A-MLP conference, this article recommends that as they work to maximize their health justice impact, A-MLPs should (A) engage intentionally in anti-racist work, (B) pursue structural reforms of health and justice systems, (C) re-envision how law and medical systems engage with communities and build their power, (D) leverage their educational reach to include more learners and increase the depth and breadth of experiences, and (E) provide leadership around MLP research and evaluation.

#### A. Anti-Racist Work in Pursuit of Racial Justice

Despite primarily serving communities of color, MLPs have historically focused on poverty as the root of health injustice rather than explicitly addressing racism and health equity.^46^ Especially in light of the perpetuation of racial inequities by legal and medical institutions, without explicitly working to combat structural racism, MLPs may unintentionally “serve to uphold and legitimize the structures that maintain institutional racism.”^47^ To avoid such outcomes, A-MLPs must consider how to structure their teaching, service to the community, and research to advance racial justice. These considerations include exploring the role of racism in the social and structural components of health, and the ways in which racism itself harms health, both of which are critical components of health injustice.^48^ Medha Makhlouf, Dina Shek, and other A-MLP scholars have urged the development of explicit racial justice strategies in MLP work, such as teaching and advocacy grounded in principles of critical race theory.^49^

A-MLPs working to address the racist impacts of the social and structural determinants of health, such as health harms rooted in racial injustices in housing and K-12 education,^50^ can also address racism in their own disciplines and professions. Interprofessional education is an important approach to teaching students about racism in the context of healthcare^51^ and how to address racism in the legal profession and legal systems. A-MLPs can similarly use interprofessional education to encourage future health professionals and lawyers to consider the role of implicit bias in provider-patient and attorney-client relationships and think critically about the ways in which both their own practices and structural racism in legal and health systems can disempower patient-clients and maintain systems of racial insubordination and white supremacy.^52^ A-MLPs should build on student advocacy urging legal and medical institutions to respond in concrete ways to the injustices of historical and ongoing racism.^53^

#### B. Structural Reform Efforts in Pursuit of Health Justice

Another strength of many A-MLPs is their potential to have a broader structural impact on health justice. The MLP patients-to-policy approach involves moving beyond individual patient/client encounters to affect change at the population health/systems levels through law and policy reform advocacy on multiple levels.^54^ Through this approach, MLPs use their individual client advocacy to recognize problems and patterns with the law and then harness that information to guide efforts to achieve broader solutions.^55^ A-MLPs can increase this impact by training law and health professions students to listen to the concerns of clients and community members and to identify systemic problems that necessitate law and policy reform.^56^

For example, law and medical students participating in the HJA Law Clinic have helped families living in housing that exposes their children to lead, which can have significant long-term developmental and health impacts. In addition to their individual case work, these students partnered with physicians and clients to testify in support of a bill to strengthen Washington, DC’s lead prevention and remediation laws. The experience highlighted the unique perspective, credibility, and advocacy power that doctors and clients with lived experience can bring to such law reform efforts.^57^ In addition to advancing their advocacy skills, when students engage in such patients-to-policy systemic advocacy, they develop “nonbiologic determinants of health, cultural diversity, public health and prevention, and health policy and systems thinking,” that they can take into their future practices.^58^ Thus, deepening and increasing collaborative policy advocacy by A-MLPs has the benefit not only of supporting systemic change around immediate issues, but also of training future leaders in law and medicine to pursue the types of population health efforts and structural reforms needed to advance health justice.

#### C. Reenvisioning How Law and Medical Systems Engage with Community

Another area for A-MLP growth is client and community engagement to support structural change. More research and the development of best practices are needed so that A-MLPs can build the power of affected individuals and communities to drive structural reform and health justice advocacy agendas. To that end, A-MLPs are exploring how to cede power to clients and communities and become humble resource allies who support such efforts.^59^ Dina Shek has argued that MLPs should shift away from traditional top-down client approaches and toward community-led justice efforts like “community lawyering” and “rebellious lawyering.”^60^ To challenge institutional racism and promote self-advocacy of marginalized communities, Shek’s A-MLP, MLP Hawai’i, has intentionally worked to decrease staff presence and role and to shift the balance of knowledge and decision-making toward those most affected so that staff and community members are working as equals.^61^ Similarly, the Loyola Health Justice Project’s Health Justice Lab includes a community-focused advocacy project where interprofessional student groups partner with community leaders to listen to their concerns and goals and work collaboratively on health issues like water affordability and contaminated soil.^62^ The Association of American Medical Colleges (AAMC) Center for Health Justice has joined the call for deeper community engagement as a way to build population health.^63^

A-MLPs also teach how justice and health can be *brought to* communities that experience unmet legal needs and health disparities.^64^ A-MLPs allow aspiring lawyers to practice in a model that removes typical barriers for marginalized and minoritized people to access legal assistance, such as difficulty navigating legal resources, transportation and childcare barriers, stigma, and a lack of trust of legal systems.^65^ By reaching people in familiar healthcare settings, A-MLPs train law students to go out into the community in a model that increases access to justice.^66^ Similarly, health professions students experience how they can advance health and health equity by facilitating access to justice through integration of legal resources into practice settings.

#### D. Extending Educational Reach and Longitudinal Exposure

As the A-MLP movement evolves, greater efforts to teach pre-professional learners across disciplines is one way to expand the model’s impact. Interprofessional education on the healthcare side, for example, is typically limited to health-related learners (e.g., doctors, nurses, dentists, pharmacists, social workers, and other allied health professionals). Other than in the A-MLP context, it does not include lawyers. In legal education, interprofessional efforts are even more limited; outside experts are generally engaged for very specific or technical expertise. A-MLPs present multiple options for breaking down educational silos and broadening academic perspectives about interprofessional education. Ultimately, A-MLPs may help normalize the expectation that law and healthcare professionals need to collaborate as part of their primary work.

Another step for A-MLPs to consider is more explicit rejection of the “medical” and “legal” framing of the model’s title. Both fields should strive to invite learners in areas such as public health, business, communications, public policy, computer science, and others to join A-MLPs and promote the roles they can play in advancing health justice. By connecting A-MLP opportunities to existing learning objectives and competencies in relevant academic disciplines, A-MLPs can drive institutional change needed to foster and support innovative approaches to interprofessional education.^67^

A further area for A-MLP growth is integration of learners at additional stages of education as a way to increase longitudinal exposure and learning. Maximizing impact on learners of a complex interprofessional practice model requires more than a single “dose” of MLP exposure. Thus, as A-MLPs evolve, they should embrace and deliberately create multiple learning opportunities for students over time. In the healthcare context specifically, A-MLPs should seek to contribute to education across the curriculum and at different stages of learning.^68^ Currently, preclinical medical education seems to be the default period for imparting innovative concepts and knowledge around health related social, political, environmental, economic, and other issues. Even where students have access to A-MLP learning in this space, it is often in elective form, which limits its capacity to shift students’ perspectives. The HJA and other A-MLPs are exploring opportunities throughout the four-year undergraduate medical, residency, and fellowship periods to address this gap. Ideally, these efforts will coincide with changes in medical education objectives and competencies aimed at graduating doctors who are able to serve as physician advocates and interprofessional collaborators ready to transform the hospitals and healthcare systems where they work.

In terms of legal education, the majority of A-MLP learning is reserved for upper-level students who may select from seminars, externships, research, and law clinic opportunities. While the standard law school curriculum greatly limits the ability to introduce first-year students to A-MLP, possibilities include electives if they are offered, and pro bono or other volunteer programs. Another area that is ripe for longitudinal growth is in legal fellowships for new law graduates so they can continue their MLP work and hone their skills as MLP attorneys within the A-MLP model. The HJA, for example, is currently hosting three HJA Law Clinic alumni as post-graduate public interest law fellows through Equal Justice Works.^69^

Finally, to the extent feasible, A-MLPs should consider how to introduce undergraduate students and even pre-undergraduate learners to the MLP model. A-MLP exposure rarely occurs before law, medical, and other health professions students have already committed to specific career paths. Providing students with an MLP perspective before they decide to pursue a particular profession would give them time and context to consider different health and law related pathways to meet their career goals. Overall, any effort by A-MLPs to expand the type and level of students who learn or participate in MLP work will enhance their educational impact.

#### E. Expanding the Next Generation of Research and Evaluation Efforts

The empirical evidence of MLP impact on a variety of individual, provider, and systems-level outcomes is growing. Room exists, however, for A-MLP scholars to champion research to address unanswered questions as the movement expands. The next generation of research and evaluation should consider how to measure whether A-MLP teaching and practice helps shift racial equity and other important outcomes. As A-MLPs expand their scope from patients to policy, researchers must also find ways to measure A-MLP impact on legislative and regulatory changes at the local, state, and federal levels.

Centering the lived experiences of clients in the research and evaluation work of A-MLPs parallels the work needed to build the power of communities in a more deep and meaningful way. Partnering with researchers who embrace community-based participatory approaches and community-engaged research paradigms will be essential in providing those with lived experience opportunities to define the outcomes they care most about. These approaches build the power of communities and center their priorities in future studies and in A-MLP educational, service, and policy advocacy efforts.^70^ And finally, there is a need for more robust studies of the impact of MLPs on a variety of student learners–beyond law and medicine–as well as a need to capture the longitudinal effects that this type of interprofessional experience can have from undergraduate through postgraduate training.

The potential to influence how future health and legal professionals practice and understand and advance health justice is a key area of growth and influence for A-MLPs nationally. As the A-MLP movement continues to evolve, identifying broad objectives and the range and types of activities available to meet them will help schools of law, medicine, nursing, public health, social work, and other disciplines maximize these opportunities. Many of the issues and themes described above are among those around which the field is beginning to coalesce; they offer exciting new ways to think about the future work of A-MLPs in advancing health justice. Ultimately, the vision for graduates and new partnerships emerging from the A-MLP movement is for them to leverage the combined power of law and medicine on behalf of individual patients and clients; to effect even broader change through community power-building and disruption, dismantling, and transformation of systems that drive health inequities; and to advance legislative, regulatory, and policy changes to make both health and legal systems more just.

A critical examination of the current evidence base for the effectiveness of MLPs raises some important challenges to the next generation of scholars seeking to spread and scale the model. For example, while randomized clinical trials are often seen as the most compelling form of evidence to policy- and decision-makers, they raise ethical concerns about withholding legal services from patients who have identified needs, given the evidence that these services are effective. There are also questions about which outcomes should be measured and to whom the benefits should be conferred. Conversations about “return on investment” have intuitive appeal but quickly become complicated. Some MLP studies report on the financial benefits that accrue to the recipients of legal services; but health systems may prefer to see those returns on their investment in the form of savings to the healthcare system. Differences between cost savings and increased revenues to the healthcare system are also important to parse when making the case for investing in an MLP.^71^ Good arguments may also exist for abandoning the notion of return on investment as a measure of doing what is necessary to promote the health and well-being of all citizens.

A-MLPs would also benefit from adoption of common tools to measure impact on learners, clients and systems, as championed by the AAMC and the Centers for Disease Control and Prevention in the Accelerating Health Equity, Advancing through Discovery (AHEAD) Initiative.^72^ Through a partnership with three well-established MLPs, this team created a set of measurement tools and a logic model that could support future multi-site collaborations. Specifically, the tools were designed to systematically measure outcomes in three domains: 1) changes in knowledge for clinical providers, medical students, and residents/fellows who train and work in an MLP setting, 2) patient and community health outcomes, and 3) cost savings/return on investment for the healthcare systems. One recent multi-site qualitative study gathered interview data from 18 patients and 78 MLP personnel across 10 states, looking at impact at patient, provider and systems levels.^73^ As A-MLPs move forward with their efforts to deepen the evidence base for their educational, service and policy work, adopting common tools and a multi-site design can increase the power and reach of their findings.

## Conclusion

The potential to influence how future health and legal professionals practice, understand, and advance health justice is a key area of growth and influence for A-MLPs nationally. As the A-MLP movement continues to evolve, identifying broad objectives and the range and types of activities available to meet them will help schools of law, medicine, nursing, public health, social work, and other disciplines maximize these opportunities. Many of the issues and themes described above are among those around which the field is beginning to coalesce; they offer exciting new ways to think about the future work of A-MLPs in advancing health justice. Ultimately, the vision for graduates and new partnerships emerging from the A-MLP movement is for them to leverage the combined power of law and medicine on behalf of individual patients and clients; to affect even broader change through community power-building and disruption, dismantling, and transformation of systems that drive health inequities; and to advance legislative, regulatory, and policy changes to make both health and legal systems more just.
